# The common causes leading to pancytopenia in patients presenting to tertiary care hospital

**DOI:** 10.12669/pjms.295.3458

**Published:** 2013

**Authors:** Kirpal Das Makheja, Bharat Kumar Maheshwari, Shafique Arain, Suneel Kumar, Sangeeta Kumari, Vikash

**Affiliations:** 1 Dr. Kirpal Das Makheja, Senior Registrar, Jinnah Postgraduate Medical Center (JPMC), Karachi, Pakistan.; 2 Dr. Bharat Kumar Maheshwari, Medical Officer, Jinnah Postgraduate Medical Center (JPMC), Karachi, Pakistan.; 3 Dr. Shafique Arain, Registrar, Jinnah Postgraduate Medical Center (JPMC), Karachi, Pakistan.; 4 Dr. Suneel Kumar, Registrar, Jinnah Postgraduate Medical Center (JPMC), Karachi, Pakistan.; 5 Dr. Sangeeta Kumari, Medical Officer, Jinnah Postgraduate Medical Center (JPMC), Karachi, Pakistan.; 6 Dr. Vikash, Medical Officer, Jinnah Postgraduate Medical Center (JPMC), Karachi, Pakistan.

**Keywords:** Aplastic Anemia, Bone Marrow Biopsy, Megaloblastic Anemia, Pancytopenia

## Abstract

***Objective:*** The objective of this study was to determine the frequency of common causes leading to Pancytopenia in patients presenting to tertiary care hospital at Karachi.

***Methods:*** A total of 62 patients with the diagnosis of Pancytopenia of more than one week duration were enrolled in the study. All patients underwent a detailed medical history and full physical examination followed by blood sampling for the investigations i.e. complete blood count with peripheral film, erythrocyte sedimentation rate (ESR), Malarial parasites (MP), liver function test, Renal function tests, PT and viral profile (HBsAg, Anti-HCV), Ultrasonography of abdomen. All patients underwent bone marrow aspiration and trephine biopsy for reporting and interpretation. Duration of study was six months, from May 2010 to November 2010.

***Results:*** The average age of the patients was 37.76 ± 16.38years. Out of 62 patients, 36 (58%) were male and 26 (42%) were female. Megaloblastic anemia was the commonest cause that was observed in 41.9% cases followed by acute myeloid leukemia 27.4%, aplastic anemia 19.4% and erythroid hyperplasia 11.3%.

***Conclusion*** *:* This study concluded that most common cause of pancytopenia is Megaloblastic anemia, followed by acute myeloid leukemia and aplastic anemia. Bone marrow examination is a single useful investigation which reveals the underlying cause in patients with pancytopenia.

## INTRODUCTION

Pancytopenia is the simultaneous presence of anemia, leucopenia and thrombocytopenia. Therefore it exists when Hemoglobin (Hb) is less then 13.5g/dl in males or 11.5g/dl in females; the leucocytes count is less then 4x10^3^/l and the platelets count is less than 150x10^3^/l.^1^ Initially, mild impairment in marrow function may go undetected and Pancytopenia may become apparent only during times of stress or increased demand (e.g., bleeding or infection). Varieties of hematopoietic and non-hematopoietic conditions manifest with features of pancytopenia. The underlying mechanisms are: decrease in hematopoietic cell production, marrow replacement by abnormal cells, suppression of marrow growth and differentiation, ineffective hematopoiesis with cell death, defective cell formation which are removed from the circulation, antibody mediated sequestration or destruction of cells and trapping of cells in a hypertrophied and over active reticuloendothelial system.^2^^,^^3^

The commonest clinical manifestations of Pancytopenia are usually Fever (86.7%), fatigue (76%), dizziness (64%), weight loss (45.3%), anorexia (37.3%), night sweats (28%), pallor (100%), bleeding (38.7%), splenomegaly (48%), hepatomegaly (21.3%), and lymphadenopathy is (14.7%).^4^

Bone marrow examination is extremely helpful in evaluation of Pancytopenia.^5^ This allows complete assessment of marrow architecture and the pattern of distribution of any abnormal infiltrate and for the detection of focal bone marrow lesions.^6^^,^^7^ While bone marrow failure syndromes and malignancies are important causes, certain non-malignant conditions such as infection and nutritional anemia are equally important causes.^5^ The most common causes leading to Pancytopenia on Bone Marrow examination are Hypoplastic (AA) bone marrow (29.05%), Megaloblastic anemia (MA) (23.64%), Hematological malignancies i.e. Acute Myeloid Leukemia (AML) (21.62%), and Erythroid hyperplasia (EH) (19.6%).^8^

The Megaloblastic anemia as a cause of pancytopenia falls in the wide range of the results reported in local studies that vary from 38% to 72%.^1^^,^^6^^,^^9^ Yet it is a useful technique not only in the diagnosis of different blood disorders but also for various systemic illnesses including pyrexia of unknown origin (PUO). Metastatic involvement with tumors,^10^ granulomatous diseases,^11^ storage disorder,^12^ haemophagocytic syndrome,^13^ and Leishmaniasis.^14^^,^^15^ This study was conducted to see any change in trends of common causes leading to Pancytopenia and the change in magnitude of common causes leading to Pancytopenia. The findings would be useful to make strategies to overcome these common causes.

## METHODS

This was a cross-sectional study carried out in the Department of Medicine, Jinnah Postgraduate Medical Centre Karachi between May 2010 to November 2010. We included adult patients of both sexes having age 13 years and above. Other criteria for inclusion were persistent Pancytopenia on peripheral blood film of more than one week duration & patients or attendants who consented for admission and bone marrow biopsy. Patients who were diagnosed cases of Malignancy, AA or Bleeding Disorder, cases of decompensated chronic liver disease, genetic causes of Pancytopenia and pregnant females with pancytopenia were excluded.

A written informed consent was obtained from all the patients after having fully explained the purpose and protocols of the study as well as risk to the patients. All patients underwent a detailed medical history and full physical examination followed by blood sampling for the investigations i.e. complete blood count with peripheral film, erythrocyte sedimentation rate (ESR), malarial parasites (MP), liver function test, renal function tests, PT and viral profile (HBsAg, Anti-HCV), ultrasonography of abdomen. After taking all the aseptic measures and with standard technique the diagnostic bone marrow aspiration and trephine biopsy were done from posterior iliac crest under adequate local anaesthesia by using Salah and Jamshidi needles, respectively. Bone marrow aspirate smears were prepared directly on the slides at the time of procedure and air dried. Touch imprints of trephine biopsy were made as an adjunct to biopsy which was preserved in 10% buffered neutral formalin and sent for reporting. Blood for the Complete Blood Count was collected at the same time from suitable vein after cleaning the site, vacutainer containing tripotassium salts of ethylene diamine tetra acetic acid (K3-EDTA).

Data was entered and analyzed in statistical software (SPSS-16). Frequency and percentage were computed for categorical variables like age groups, gender, duration of illness, common causes leading to pancytopenia. Mean(± SD), 95% confidence interval were computed for quantitative measurement like age, duration of illness. Stratification was done with regard to age, gender, duration of pancytopenia to see the effect on outcome.

## RESULTS

There were 62 patients with pancytopenia which were included in this study. There were 36 (58%) males and 26 (42%) females with a with 1.38:1 male to female ratio and a mean age 37.76 years ± 16.38 SD. Average duration of pancytopenia was 16.89 ± 7.09 days (Range 7 to 37 days). Duration of disease of 15 patients (24%) was below ten days, 32(52%) had tolerated disease from 11 to 20 days and 15(24%) had tolerated from above 20 days. 

**Fig.1:**
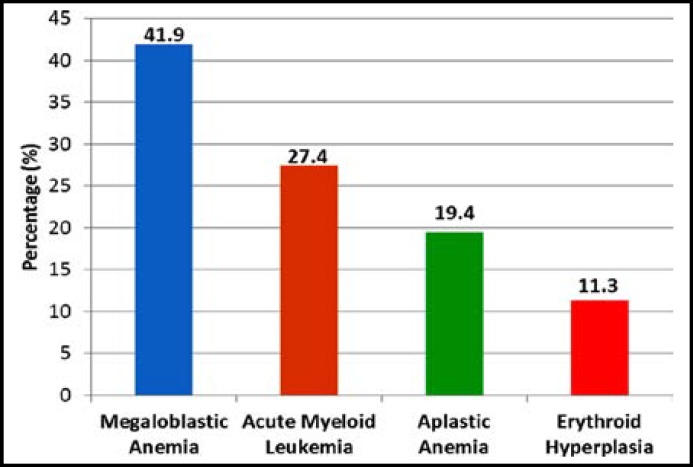
Frequency of Common Causes of Pancytopenia

Frequency of common causes leading to pancytopenia are shown in Fig.1. Megaloblastic anemia was the commonest cause that was observed in 41.9% (26/62) cases followed by acute myeloid leukemia 27.4% (17/62), aplastic anemia 19.4% (12/62) and Erythroid hyperplasia 11.3% (7/62). We did not performed levels of Vitamin B 12 & serum folic acid therefore the cause of megaloblastic anemia could not be explored. Insignificant findings were found regarding ESR, MP, LFTs, renal function tests, PT & viral profile (HBsAg, Anti-HCV) and US of abdomen.

Stratification of age groups and duration of disease are presented in Table-I to observe causes leading to pancytopenia which showed that Megaloblastic anemia was most common in 41 to 50 years however the distribution of disease among both genders were comparable as shown in Table-II.

**Table-I:** Frequency of age groups and duration of disease. (n=62)

*Age Groups*	*n*	*Common causes leading to pancytopenia*			
		*M.A*	*AML*	*A.A*	*E.H*
*13 to 20 Years*	16	7 (43.8%)	6 (37.5%)	3 (18.8%)	0 (0%)
*21 to 30 Years*	17	6 (35.3%)	3 (17.6%)	6 (35.3%)	2 (11.8%)
*31 to 40 Years*	16	7 (43.8%)	4 (25%)	2 (12.5%)	3 (18.8%)
*41 to 50 Years*	09	6 (66.7%)	1 (11.1%)	1 (11.1%)	1 (11.1%)
*51 to 60 Years*	04	0 (0%)	3 (75%)	0 (0%)	1 (25%)

M.A= Megaloblastic anaemia,

AML= Acute Myeloid Leukemia

A.A= Aplastic anaemia,

E.H= Erythroid Hyperplasia

**Table-II:** Frequency of pancytopenia among both genders. (n=62)

*Gender*	*n*	*Common causes leading to pancytopenia*			
		*M.A*	*AML*	*A.A*	*E.H*
*Male*	36	15 (41.7%)	10 (27.8%)	7 (19.4%)	4 (11.1%)
*Female*	26	11 (42.3%)	7 (26.9%)	5 (19.2%)	3 (11.5%)

M.A= Megaloblastic anaemia.

AML= Acute Myeloid Leukemia

A.A= Aplastic anaemia,

E.H= Erythroid Hyperplasia

## DISCUSSION

Pancytopenia is a common hematological problem encountered in clinical practice, which has multiple causes and the underlying pathology determines the management and prognosis of the patients. The evaluation of the cause of pancytopenia starts from history, physical examination and various laboratory investigations including basic hematological, biochemical, radiological, and histopathological investigations.

Bone marrow examination is simple and safe invasive procedure, which causes a moderate discomfort and can be performed easily. Its great utility is for investigating and it is an important diagnostic modality for evaluating the cases of pancytopenia.

Megaloblastic anemia was considered the most common cause of Pancytopenia (41.9%) in our study. Similar results have been found by other local studies.^1^^,^^6^^,^^9^^,^^16^ The diagnosis of Megaloblastic anemia in our study was established by characteristic bone marrow findings. The high prevalence of nutritional anemia in India has been cited for the increased frequency of Megaloblastic anemia. Because of geographical and social similarities, nutritional anemias may also be responsible for increased frequency of Megaloblastic anemia in northern region of Pakistan. Among the nutritional anemias Vit B12 deficiency is more prevalent than folate deficiency in Pakistan^17^and our study reported similar results. Investigation of the cause of megaloblastic anaemia was beyond the scope of this study.

The approximately comparable series of pancytopenia is from Tariq Aziz et al,^18^ Iqbal et al^19^, Qazi et al.^20^ In all these studies, Megaloblastic anemia was found to be the major cause of pancytopenia. Increased incidence of Megaloblastic anemia in these studies was probably due to high prevalence of nutritional anemia in the non-industrialized world.

Our study showed that peak incidence of Megaloblastic anemia in middle age group (41 to 50 years) with an equal sex ratio which is supported by studies.^21^^,^^22^ In contrast, Khanduri et al found in their study that the peak incidence was seen in the age group of 10–30 years (48% of patients) and there was a preponderance of women (71%). ^23^

Studies in Philippines^24^ and Nepal^25^ reported that males were affected with Aplastic anemia much more frequently then females, which might be a result of higher incidence of occupational exposure to chemicals and of pesticides exposure as a common etiological agent for Aplastic anemia in these countries. This is contrary to our study in which males and females were equally affected with Aplastic anemia. This can be explained in part by the fact that easy availability of over the counter medications and also Pakistan is an agricultural country; pesticides may be an important factor in the incidence of Aplastic anemia as equal environmental exposure to these pesticides to either sex. This variation in the frequency of etiology and other features among the studies possible due to the broad spectrum of etiologies or disorders behind pancytopenia.

## CONCLUSION

Most common cause of pancytopenia is Megaloblastic anemia. While other common causes are Acute Myeloid Leukemia, Aplastic Anemia, and Erythroid Hyperplasia.In Pakistan probably poverty, poor eating habits, poor quality of foods, and self avoidance of necessary foods may be the causes of nutritional deficiencies leading to Megaloblastic anemia.Bone marrow examination is a single useful investigation which reveals the underlying cause and prognosis in patients with pancytopenia. Megaloblastic anemia of either cause can be prevented by improving the nutritional status of our population.
